# Comparative evaluation of arresting proximal caries in primary molars with Resin Infiltration and Silver Diamine Fluoride: A Randomized Controlled Trial

**DOI:** 10.21142/2523-2754-1404-2026-314

**Published:** 2026-10-01

**Authors:** Aishwarya Kamble, Devendra Patil, Farhin Katge, Vamsi Krishna Chimata, Parin Bhanushali, Shilpa Shetty

**Affiliations:** 1 Department of Pediatric and Preventive Dentistry, Terna Dental College. Navi-Mumbai, Maharashtra, India. aishwaryakamble2394@gmail.com, dev1987endra@gmail.com, pedotdc@gmail.com, kc.vamsi@gmail.com , dr.parin91@hotmail.com, shilpakarthick@gmail.com Department of Pediatric and Preventive Dentistry Terna Dental College Navi-Mumbai Maharashtra India aishwaryakamble2394@gmail.com dev1987endra@gmail.com pedotdc@gmail.com kc.vamsi@gmail.com dr.parin91@hotmail.com shilpakarthick@gmail.com

**Keywords:** silver diamine fluoride, dental caries, primary dentition, resin cements, fluoruro de diamina de plata, caries dental, dentición primaria, cementos de resina

## Abstract

**Introduction:**

Proximal caries are considered as a challenge to control in primary molars. Resin infiltration and silver diamine fluoride are non-invasive measures to arrest proximal caries. The objective of this study was to evaluate and compare arrest of proximal caries in primary molars between resin infiltration and silver diamine fluoride. The secondary objective is to assess any difference in dental anxiety experienced by the patient during both treatment approaches.

**Methods:**

A split mouth randomized controlled trial included forty children aged 4 to 10 years with a total of 140 primary molars in both maxillary and mandibular arches. The study population was randomly divided into two groups: Group A - Resin infiltration and Group B - SDF with the help of computer assisted randomization. Follow up evaluation was done at 6 and 12 months using standardized bitewing radiographs. Dental anxiety was evaluated using items from Faces version of Modified Child Dental Anxiety Scale (MCDASf) questionnaire. The values were then statistical analyzed using Chi square test.

**Results:**

There was no statistically significant difference between resin infiltration and silver diamine fluoride in arrest of proximal carious lesions (p = 0.269). The comparison between resin infiltration and silver diamine fluoride of dental anxiety showed that children were significantly more anxious after the resin infiltration procedure (p = 0.009).

**Conclusion:**

Resin infiltration and silver diamine fluoride are both efficacious in arresting proximal carious lesions in primary molars although children experienced more anxiety after the resin infiltration procedure.

## RESUMEN

**Introducción:**

La caries proximal representa un desafío en el control de los molares primarios. La infiltración de resina y el fluoruro de diamina de plata son medidas no invasivas para detener la caries proximal. El objetivo de este estudio fue evaluar y comparar la detención de la caries proximal en molares primarios mediante la infiltración de resina y fluoruro de diamina de plata. El objetivo secundario fue evaluar las diferencias en la ansiedad dental experimentada por el paciente durante ambos tratamientos.

**Materiales y métodos:**

Se realizó un ensayo clínico aleatorizado con diseño de boca dividida que incluyó a 40 niños de 4 a 10 años, con un total de 140 molares primarios en ambos arcos, maxilar y mandibular. La población de estudio se dividió aleatoriamente en dos grupos: Grupo A: infiltración de resina y Grupo B: fluoruro de diamina de plata, mediante aleatorización asistida por computadora. El seguimiento se realizó a los 6 y 12 meses a través de radiografías interproximales estandarizadas. La ansiedad dental se evaluó de acuerdo con los ítems de la versión de caras del cuestionario de la Escala Modificada de Ansiedad Dental Infantil (MCDASf). Los valores se analizaron estadísticamente mediante la prueba de chi cuadrado.

**Resultados:**

No se observaron diferencias estadísticamente significativas entre la infiltración de resina y el fluoruro de diamina de plata en la detención de las lesiones cariosas proximales (p = 0,269). La comparación entre la infiltración de resina y el fluoruro de diamina de plata, en cuanto a la ansiedad dental, mostró que los niños experimentaron mayor ansiedad tras la infiltración de resina (p = 0,009).

**Conclusión:**

: Tanto la infiltración de resina como el fluoruro de diamina de plata son eficaces para detener las lesiones cariosas proximales en molares primarios, aunque los niños presentaron mayor ansiedad tras la infiltración de resina.

## INTRODUCTION

Proximal caries in primary molars presents a significant clinical challenge due to limited accessibility, larger contact areas with adjacent teeth, and reduced salivary action, all of which hinder early detection and effective management. ¹^-^³ Traditionally, management of proximal caries involves the “drill and fill” approach to gain access to the lesion; however, this often results in substantial loss of sound tooth structure. Furthermore, restored teeth may undergo repeated cycles of restoration replacement, commonly described as the “death spiral of restorations,” ultimately compromising tooth longevity. ⁴

In recent years, there has been a paradigm shift toward minimally invasive dentistry, emphasizing the preservation of tooth structure through non-invasive and micro-invasive approaches. ⁵^,^⁶ Among these, remineralizing agents such as fluoride varnish and silver diamine fluoride (SDF) have been widely used for arresting early carious lesions. SDF, in particular has demonstrated high efficacy in caries arrest; however, its major drawback is the unaesthetic black discoloration of treated lesions, which may limit its acceptance, especially in visible and proximal areas. ^7^

Resin infiltration has emerged as a novel micro-invasive technique for the management of incipient caries. ^8^ This technique involves penetration of a low-viscosity resin into the porous structure of enamel lesions via capillary action, thereby arresting lesion progression by occluding diffusion pathways. ICON® is a commercially available system based on this principle. Compared to conventional remineralization strategies, resin infiltration offers the additional advantage of improved esthetics without cavitation or removal of tooth structure. ^9^

Despite the proven effectiveness of both SDF and resin infiltration in managing early carious lesions, there is limited in vivo evidence directly comparing their efficacy specifically for proximal caries in primary molars. Additionally, while both techniques are minimally invasive, their impact on dental anxiety in pediatric patients remains an important clinical consideration that has not been adequately explored.

Therefore, the present study was undertaken to comparatively evaluate the efficacy of resin infiltration and SDF in arresting proximal carious lesions in primary molars, and to assess the level of dental anxiety experienced by pediatric patients undergoing these treatment modalities.

## MATERIALS AND METHODS

The study was designed as a split mouth randomized controlled trial and was registered at www.clinicaltrials.in.th (CTRI/2019/10/021636). The study was approved from Institutional Review Board Ethics Committee (IRB-EC) (Registration: TDC- IEC-TDC/20/2018).

### Patient selection

The study population included patients who reported to outpatient department of Pediatric and Preventive Dentistry. The procedure of study was informed to parent or guardian and informed consent was obtained for the same. It was a single blinded study where patient was unaware of the type of intervention.

### Patient Consent Statement (Declaration of Helsinki)

Written informed consent was obtained from the patient prior to participation in the study. The patient was clearly informed about the nature and purpose of the study, the procedures involved, potential risks and benefits, and their right to refuse participation or withdraw at any time without affecting their treatment.

The study was conducted in accordance with the ethical principles outlined in the Declaration of Helsinki. Confidentiality of the patient’s identity was strictly maintained, and no identifying information has been disclosed in this study.

Oral examinations were conducted under adequate dental operatory light using mouth mirror and probe according to the International Caries Detection and Assessment System (ICDAS) II criteria. ^11^ An elastic separator was used to facilitate tooth separation for correct selection of tooth. This was followed by routine bitewing radiographic examination to detect initial caries lesions which may or may not be detected on visual examination. ^12^

### Sample size calculation

Sample size calculation was done according to results from a study by Page et al. G* power software (version: 3.0.10; Germany) was used for sample size calculation. ^11^ The sample size of 140 sites were allocated in 40 subjects (p1 = 0.78, p2 = 0.57) with level of significance (alpha) set at 0.05. Power of the study considered for the present was (1− beta) 80% (0.8). 

Fifty-six patients were assessed for eligibility, out of which 40 children who met inclusion and exclusion criteria were selected for this study. A total of 140 primary molars of both maxillary and mandibular arches were included in the study. 

### Inclusion and Exclusion criteria

Four- to 10-year-old healthy children with Frankl’s behaviour rating 3 and 4 with bilateral presence of two primary molars were included in the study. Also, primary molars showing proximal carious lesions without cavitation (ICDAS codes 1-4) with radiographic scores E1, E2 or D1 on bitewing radiographs were included in the study. Primary molars near to exfoliation stage (less than two years), proximal cavitated lesions (ICDAS codes 5 & 6) or primary molars in contact with adjacent restored proximal surface were excluded from the study. The exclusion criteria also included un-cooperative patients with Frankl’s behaviour rating 1 and 2.

### Procedure 

The samples were randomly divided in Group A (Resin infiltration) and Group B (SDF). The randomization was done using computer assisted randomisation using software Rando 1.2 version 2004 (JIPMER, Pondicherry). Allocation concealment was done using sealed opaque envelopes. Proximal surfaces of selected primary molars were cleaned using a slow speed micromotor handpiece, pointed bristle brush and prophylactic paste. The teeth selected for study were then rinsed with water and air dried using three-way syringe. 

#### Group A: (Figure 1)

Rubber dam isolation (Hygenic Corporation, USA) was carried out under topical anaesthesia (Lox 2%, Neon Laboratories Ltd., India) and local anaesthesia using lignocaine with adrenaline 1:80,000 (Lignox 2% A, Indoco Remedies Ltd., India). Mandibular and maxillary primary molars were anaesthetized using infiltration technique. A wedge was placed between the primary molar and adjacent tooth to create space for placement of proximal applicator tip. Etching of interproximal surfaces of selected primary molars was done with 15% hydrochloric acid for 120 seconds. Etchant was applied using unique proximal applicator tip was provided in the ICON proximal kit (DMG, Germany). Then teeth were rinsed with water and air dried using three-way syringe. Ethanol (99%) was then applied with a syringe for 30 seconds. ICON® (DMG, Germany) resin infiltrant was then applied on affected interproximal surface using the proximal applicator tip. The whole procedure was done according to the manufacturer’s instructions. The final stage of curing was done for 120 seconds using light curing unit (Ivoclar Vivadent Marketing Ltd, USA). Reapplication of resin infiltrant was done with polymerization for 60 seconds. Post-operative instructions were given to the patient.

**Figure 1:**
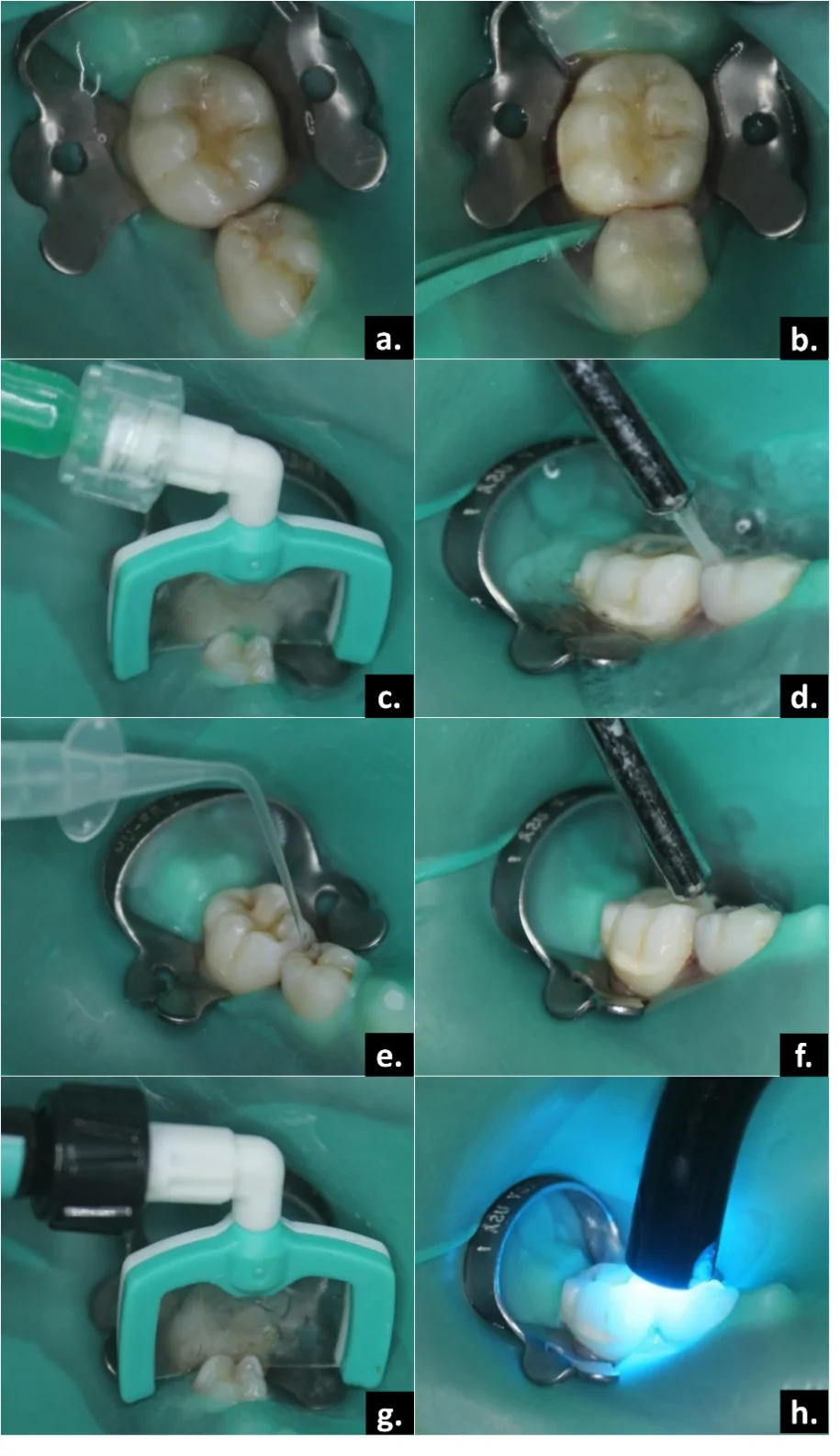
Procedure for Group A - Resin Infiltration: a) Rubber dam application; b) Wedge placement; c) Etching with 15% hydrochloric acid; d) Rinsing with water; e) 99% ethanol; f) Air drying; g) Resin infiltration (ICON®; Hamburg; Germany); h) Curing.

#### Group B: (Figure 2)

Vaseline was coated on lips and surrounding gingival tissues to avoid staining. Rubber dam isolation, local anaesthesia administration and wedge placement was done as described in Group A. SDF (Fagamin, Argentina) was applied using applicator tip for one minute to affected interproximal area. Unwaxed dental floss was used to disperse SDF uniformly into the interproximal surfaces and was kept isolated for three minutes. Post-operative instructions were given to the patient.

**Figure 2:**
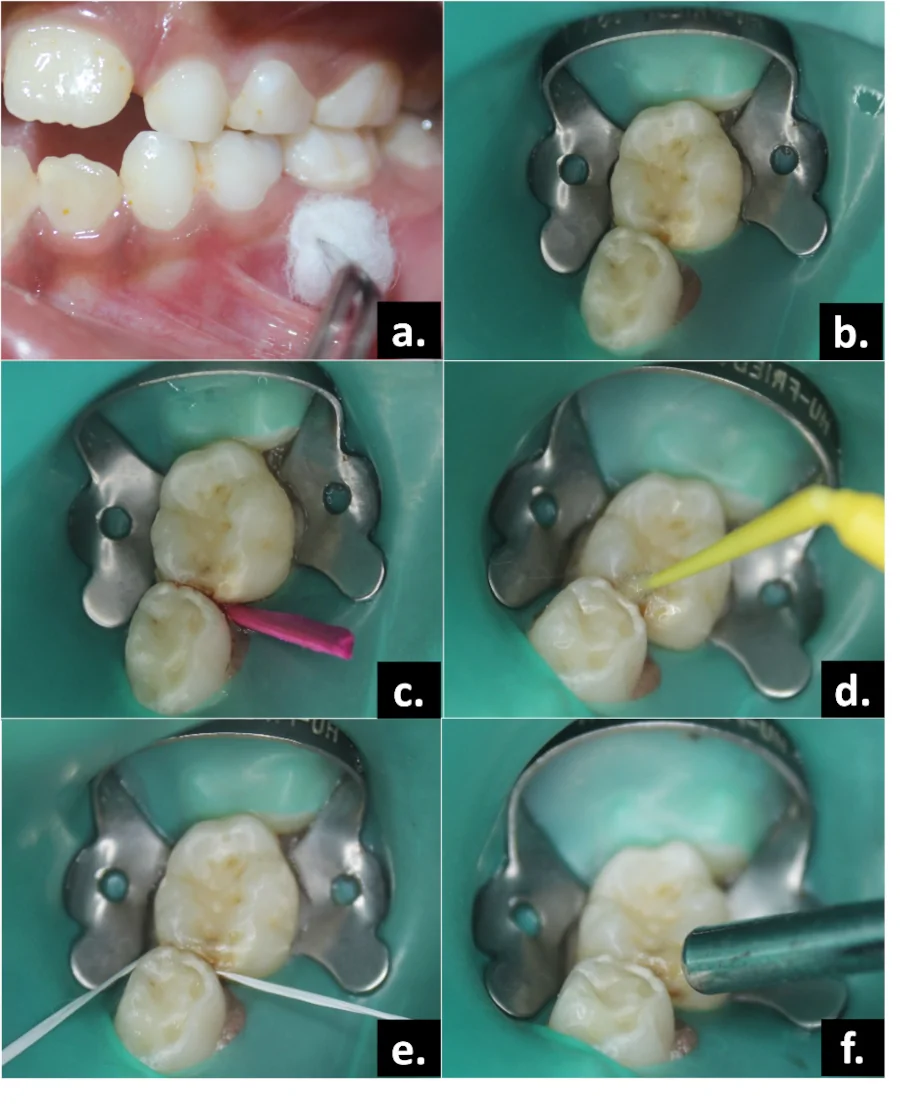
Procedure for Group B - Silver Diamine Fluoride: a) Application of Vaseline on surrounding gingiva; b) Rubber dam isolation; c) Wedge placement; d) SDF application (Fagamin; Argentina); e) Use of dental floss; f) Air drying.

### Evaluation

Follow up evaluation was done at six and 12 months using standardized bitewing radiographs by two independent examiners. Both the examiners were independently blinded to treatment group allocation. At follow up intervals, any carious lesion proceeded to D3 or any visual cavitation received restoration. Radiographic scores were given to classify the depth of proximal carious lesions which was given by Mejare et al. ^13^

Before and after treatment, Dental anxiety was evaluated using items from Faces version of Modified Child Dental Anxiety Scale (MCDASf) questionnaire. ^14^ The scale was graded according to following responses:

1. Relaxed, not worried
2. Very slightly worried
3. Fairly worried
4. Worried a lot
5. Very worried

Each response in MCDASf was accompanied by appropriate smiley face. The children in present study were asked to answer the following: “Was it alright while application of resin infiltration or SDF?”, “Was the procedure painful?” and “Would they like another tooth to be treated with resin infiltration or SDF?”. 

### Statistical analysis

The radiographic scores for caries progression within and between the groups were analysed using Chi-square tests. The mean Faces scores were also calculated with chi-square test to determine changes in anxiety level before and after the procedure. All testing was done using two-sided tests at alpha 0.05. The confidence interval was set to 95% and p < 0.05 was considered statistically significant. Inter examiner and intra examiner reliabilities were calculated using interclass correlation coefficient (ICC). Data was analysed using windows based ‘Statistical Package for the Social Sciences’ statistics software (SPSS software, version 18).

## RESULTS

### Baseline

In the present study, 40 children including 18 girls and 22 boys were selected. The mean age of children was 6.8 (±1.29) ranging from 4 to 10 years. Tables 1 and 2 show the distribution of teeth and radiological outcome values at baseline, six months and 12 months follow-up. The caries risk assessment was recorded based on AAPD guidelines with 4 children at high risk (10.3%), 24 with moderate risk (61.5%) and 11 with low risk (28.2%). The consort diagram shows the sample size distribution and dropouts at baseline, six months and 12 months follow-up. (Figure 3) 

**Table 1:** Radiographic score distribution of samples at baseline, 6 months and 12 months

	GROUP A			GROUP B		
	Baseline	6 Months	12 Months	Baseline	6 Months	12 Months
E1	28 (40%)	21 (30%)	20 (28.6%)	30 (42.9%)	26 (37.1%)	21 (30%)
E2	29 (41.4%)	33 (47.1%)	29 (41.4%)	27 (38.6%)	28 (40%)	24 (34.3%)
D1	13 (18.6%)	10 (14.3%)	5 (7.1%)	13 (18.6%)	11 (15.7%)	9 (12.9%)
D2	-	1 (1.4%)	1 (1.4%)	-	-	3 (4.3%)

**Figure 3:**
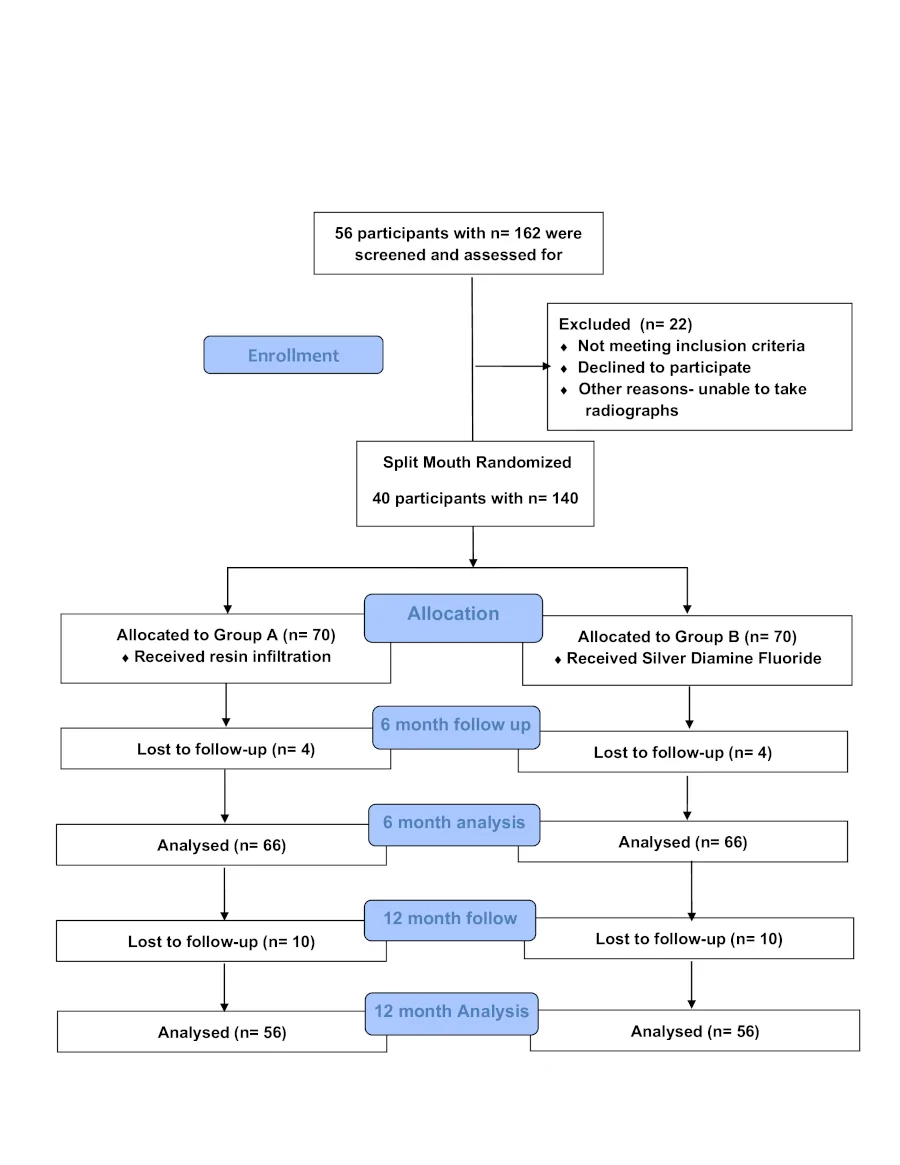
Flowchart of the full sample at baseline, 6 months and 12 months according to CONSORT guidelines.

On radiographic examination, at baseline, caries was present in 58 teeth (82.9%), restricted to outer half of enamel (E1). In 56 teeth (80%), caries was restricted to the inner half of enamel extending upto but not beyond the dentino-enamel junction (E2) whereas in only 26 teeth (37.2%) caries was involving the dentin just beyond the dentino-enamel junction (D1).

### Progression

The radiographic score after six months showing progression from E1 to E2 was seen in four teeth in Group A whereas one tooth showed same progression in Group B. (Table 2) One tooth each in Group A and Group B with radiographic score of D1 progressed to D2. (Figures 4 and 5)

**Table 2:** Caries progression after 6 months and 12 months in Group A and B

	6 months			12 months		
	Group A	Group B	Pearson Chi-Square	p value	Group A	Group B	Pearson Chi-Square	p value
No Progression	60 (90.9%)	64 (97.0%)	2.129	0.274	55 (91.7%)	50 (83.3%)	1.905	0.269
Progression	6 (9.1%)	2 (3.0%)			5 (8.3%)	10 (16.7%)		

Pearson Chi-Square test

**Figure 4:**
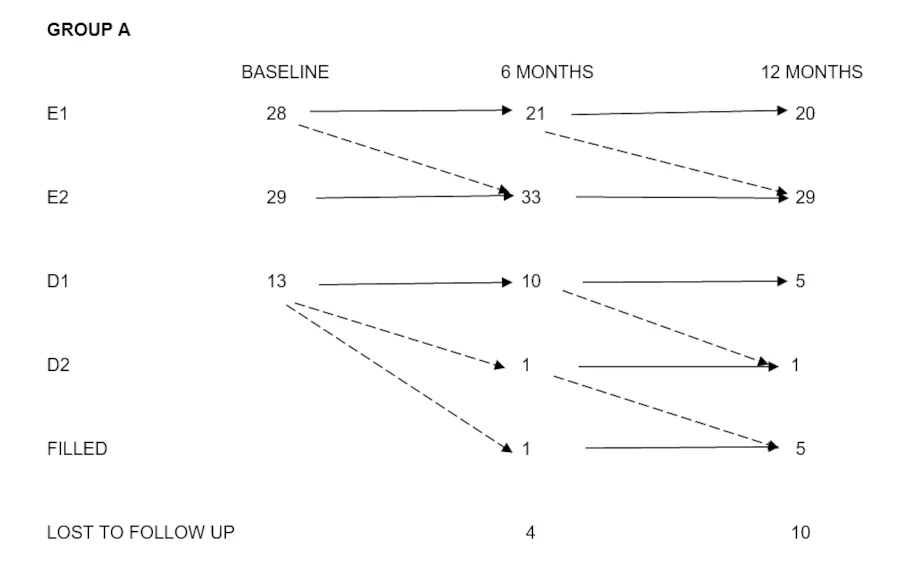
Radiographic assessment of caries progression after 6 months and 12 months in Group A.

**Figure 5:**
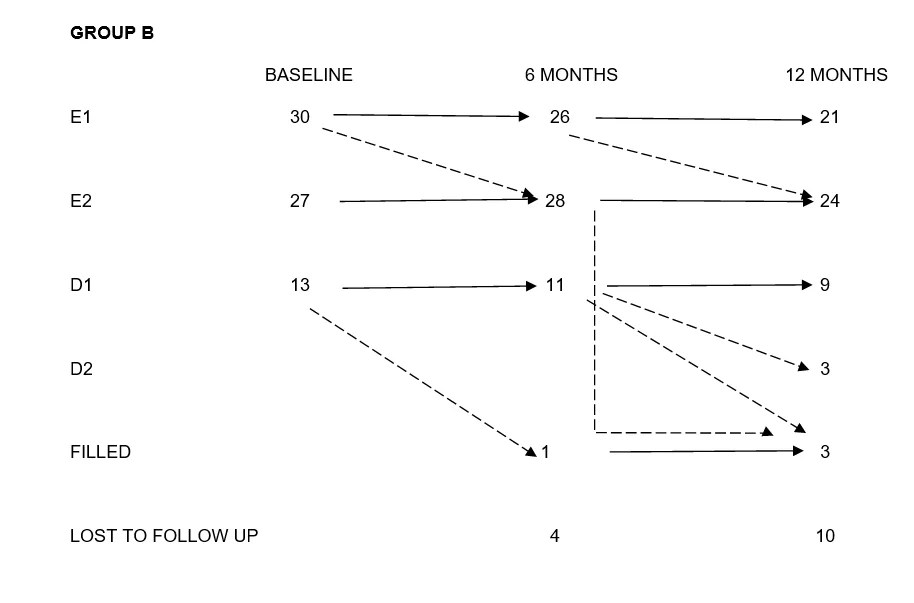
Radiographic assessment of caries progression after 6 months and 12 months in Group B.

Radiographic progression of scores at 12 months follow up showed one tooth in Group A and four teeth in Group B had progressed from E1 to E2. Whereas two teeth in Group B showed progressing carious lesions from E2 to D1. Progression from D1 to D2 was seen in one tooth in Group A and four teeth in Group B. In Group A, four teeth with D1 and one tooth with D2 were progressed to be filled whereas in Group B, two teeth initially scored as E2 had to be filled. (Figures 4 and 5)

On pairwise comparison, no statistically significant difference was found between Group A and Group B regarding proportion of caries progression at 6 months and 12 months. At 6 months, 9.1% (6/66) had progressed in resin infiltration group (Group A) whereas, in Group B, 3% (2/66) showed progression (p=0.274). Whereas at 12 months follow up, 8.3% (5/60) in Group A and 10.7% (10/60) in group B had progressed (p=0.269). (Table 3) Since, no statistically significant difference was observed in both Group A and Group B regarding proportion of caries progression at 6 months and 12 months, the null hypothesis is accepted.

**Table 3:** MCDASf scores before and after treatment for Group A and B

Modified Child Dental Anxiety Scale (Faces score)	BEFORE			AFTER			
	GROUP A	GROUP B	Pearson Chi-Square	p value	GROUP A	GROUP B	Pearson Chi-Square	p value
0	24	23	0.564	0.905	4	6	15.262	0.009*
2	8	8			8	16		
4	4	6			7	11		
6	4	3			14	4		
8	-	-			6	1		
10	-	-			1	-		

Pearson Chi-Square test

*Significant

### Dental anxiety

Before treatment, the mean scores of MCDASf for dental anxiety showed no significant difference in both Group A and B (p = 0.905). Before the treatment procedures, in both Group A and Group, 24 children (51.1%) and 23 children (48.9%) showed scored zero which was termed as “relaxed”. However, the mean MCDASf scores after treatment between both groups showed statistically significant difference (p = 0.009). In group A post treatment, the MCDASf scores selected by the children showed higher level of anxiety and discomfort. Fourteen children were fairly worried (score 6), 6 of them were worried a lot (score 8) and one was very worried (score 10). (Table 4 and Table 4) Whereas in Group B, children showed lower anxiety scores as compared to Group A. When the children or their parents were questioned directly, neither group found any related side effects such as pain, loss of vitality or any gingival damage.

**Table 4:** Within group comparison of MCDASf scores

Treatment interval	Group A			Group A	
	Pearson Chi-Square	p value	Pearson Chi-Square	p value
Before vs After	27.65	0.00004*	15.20	0.004*

Pearson Chi-Square test

*Significant

## DISCUSSION

The resin infiltration kit (ICON®, DMG, Germany) consists of an infiltrate consisting of tetraethylene glycol dimethacrylate, 15% hydrochloric acid as an acid conditioner and ethanol. ^15^^-^^17^ ICON® resin infiltration proximal kit was used in Group A and 38% SDF in Group B. 38% SDF was used instead of fluoride varnish as it contains both silver (2,53,870 ppm) and fluoride (44,800 ppm) as compared to varnish which has a lower concentration of fluoride (22,600 ppm). ^18^

This study was carried out to compare efficacy of minimally invasive options like resin infiltration and SDF to arrest proximal caries in primary teeth. In group A, 15% hydrochloric acid (HCl) gel was used in the present study. This is because it has better properties than 37% phosphoric acid gel in removing the mineralized surface layer of enamel lesions for 120 seconds. In ICON kit, 15% HCl produces a penetration depth of 58 µm, double that of phosphoric acid (25µm), penetrating to the deepest lesions, eliminating demineralized areas and preventing further caries attacks. ^15^ In the present study, tooth surface was dried using 99% ethanol. In this technique, the water in the substituted demineralized collagen matrix is replaced with increasing concentrations of ethanol. Without causing any further contraction of the interfibrillar spaces resin is penetrated in the collagen matrix, thus preventing phase separation of hydrophobic resin monomers. ^19^^,^^20^ Application of ICON resin was done to lesion surface with the help of specialised proximal tip. This was done to help resin to penetrate in matrix layer for at least three minutes. After the initial application, reapplication of resin was done to fill up gaps created after shrinkage of the material in first application. ^21^^,^^22^

In group B, SDF has an inherent drawback which causes blackish discolouration of tooth. ^23^ In the present study, the SDF application procedure, drawbacks and efficiency were explained to the parents. However, most of the parents did not have an any issue with the discolouration as they were posterior teeth and gave their consent to go ahead with the treatment. 

In the present study, most of the lesions were in the outer half (40%) and inner half of enamel (42.9%) at baseline, which was similar to results by Foster Page et al. ^11^ In this study 12.1% of lesions had progressed at 6 months and 25% at 12 months. In literature, it is an established fact that proximal carious lesions progress faster as they approach dentin. In the present study as well as in previous studies, resin infiltration and SDF both showed effective results in E2 lesions than in D1 lesions. ^11^^,^^24^ Also, when the treatment was done the treated carious lesions which got progressed to D2 (middle third of dentin) were already in D1. Some of the D1 lesions may contain radiologically or clinically undetected microcavitation, which may contribute to the increased rate of progression. Once cavitation occurs, the effectiveness of the material is compromised. ^25^ Asker et al. developed an improved micro-filled infiltration resin (MFIR) that has the same ability to penetrate micro cavitated natural carious lesions when compared to conventional resin infiltration. He also explained that MFIR-filled cavities are similar to those of flowable composite resins and may help expand the application range of resin infiltration. ^4^ Clemens et al. showed that SDF was effective in early (ICDAS 1 or 2), noncavitated (ICDAS 3 or 4) and cavitated (ICDAS 5 or 6) carious lesions. ^26^

In this study, caries arrest at 6 months follow up was 90.9% (60/66) in Group A (Resin infiltration) and 97% (64/66) in Group B (SDF). At 12 months follow up, caries arrest in Group A (Resin infiltration) was 91.7% (55/60) and 83.3% (50/60) in Group B (SDF). However, no statistically significant difference was seen in caries progression with both the groups at six months and 12 months follow up. Similarly, other studies have shown that resin infiltration is effective in preventing the progression of proximal caries in both primary and permanent teeth. ^8^^,^^11^^,^^27^^,^^28^ Although the resin-infiltrated group in this study showed successful caries arrest in lesions at one year follow-up (91.7%), progressive lesions (8.3%) were characterized by either any problem in resin polymerization or deep lesions. The results of present study also revealed that SDF arrested 83.3% proximal carious lesions at 12 months in primary molars. Zhi et al. found that SDF application along with glass ionomer cement restoration and annual application of SDF, both were effective at arresting caries. ^28^ However, semi-annual application of SDF was significantly more effective than annual application. In the present study, application of SDF was done only once at baseline and thus, this may explain a higher caries progression rate as compared to resin infiltration group at one year follow-up. A retrospective study conducted by Hammersmith et al. evaluated interproximal dental caries in paediatric patients treated with SDF. They found that in the primary dentition at 12-month follow-up, after SDF application with woven floss proximal caries were arrested. ^24^

In terms of dental anxiety measured between resin infiltration and SDF, no significant difference was found in overall mean MCDASf scores before the treatment in both Group A and Group B. Whereas, significant difference in MCDASf scores was seen after the treatment in Group B. Findings of this study showed that simple intervention like SDF application caused less discomfort in children than resin infiltration which requires a greater number of clinical steps. Although a previous study by Altarabulsi et al. ^29^ had not performed local anesthesia prior to isolation, the present study advocated the use of local anesthesia in both the groups. This decision was taken to maintain uniformity, although rubber dam may not be essential for SDF procedure. Nevertheless, despite the prior use of local anesthesia, resin infiltration technique showed high level of discomfort with the child as compared to SDF after the treatment. Similar findings were seen in a study by Mattos-Silveira et al. They concluded that SDF was more effective than resin infiltration technique in reducing the discomfort. ^30^

The findings of the present study have important clinical implications for pediatric dental practice, particularly in the management of proximal caries in primary molars using minimally invasive approaches. Both resin infiltration and SDF demonstrated comparable efficacy in arresting caries progression over a 12-month period, suggesting that either modality can be considered a viable alternative to conventional restorative treatment in non-cavitated or early lesions.

From a clinical perspective, SDF may be preferred in younger or less cooperative children due to its simplicity, shorter chairside time, and minimal technique sensitivity. It is especially advantageous in community-based settings or situations where isolation is difficult. However, the associated black discoloration remains a limitation, although in the present study parental acceptance was satisfactory for posterior teeth.

On the other hand, resin infiltration offers the benefit of maintaining esthetics while preserving tooth structure, making it a suitable option in cooperative patients where adequate isolation and moisture control can be achieved. Nevertheless, the technique-sensitive nature of the procedure and the increased number of clinical steps may contribute to higher levels of patient discomfort, as observed in the present study.

Therefore, the selection of treatment modality should be individualized based on patient-related factors such as age, behavior, esthetic concerns, and clinical feasibility, thereby enabling clinicians to adopt a more tailored and minimally invasive approach to caries management.

This is one of the few studies that compares resin infiltration with SDF using a split-mouth study design over a 12-month follow-up period. Within the limitations of the present study, both modalities demonstrated promising results for arresting proximal caries in primary molars. However, further long-term studies with larger sample sizes and objective assessment methods are recommended to validate these findings and strengthen their clinical applicability.

This article was strong due to its randomized controlled design and direct comparison between two non-invasive caries-arrest techniques. It is clinically relevant to the management of proximal caries in primary molars. However, the study had some limitations, including a short follow-up duration, a limited sample size, the inability to blind evaluators due to silver diamine fluoride (SDF) staining, and operator-dependent outcomes.

## CONCLUSION

The current study concludes that both resin infiltration and silver diamine fluoride are efficacious in controlling progression of proximal carious lesions in primary molars and can be effectively incorporated in routine restorative procedures in pediatric patients.

In aspect of dental anxiety, children experienced more anxiety after the resin infiltration procedure as compared to silver diamine fluoride procedure. Hence it can be stated that silver diamine fluoride procedure can be easily accepted by pediatric patients as compared to resin infiltration procedure. 

## References

[1] Yadav SP, Meghpara M, Marwah N, Nigam AG, Godhani S, Chalana S (2022). Association of Early Childhood Caries with Feeding, Dietary Habits, and Oral Hygiene Practices among Rural and Urban School Children of Jaipur. Int J Clin Pediatr Dent.

[2] Surendranath P, Krishnappa S, Srinath S (2022). Silver Diamine Fluoride in Preventing Caries A Review of Current Trends. Int J Clin Pediatr Dent.

[3] Splieth CH, Kanzow P, Wiegand A, Schmoeckel J, Jablonski-Momeni A (2020). How to intervene in the caries process proximal caries in adolescents and adults-a systematic review and meta-analysis. Clin Oral Investig.

[4] Askar H, Schwendicke F, Lausch J, Meyer-Lueckel H, Paris S (2018). Modified resin infiltration of non-, micro- and cavitated proximal caries lesions in vitro. J Dent.

[5] Amaechi BT (2017). Remineralisation-the buzzword for early MI caries management. Br Dent J.

[6] Geethu PN, Suvedha N, Kurien AA, Chakravarthy Y, Pallavi V (2023). Demineralization and remineralization in restorative dentistry. J Academy Dent Educ.

[7] Meligy OAESE, Ibrahim STE, Alamoudi NM (2018). Resin Infiltration of Non-Cavitated Proximal Caries Lesions A Literature Review. J Oral Hyg Health.

[8] Peters MC, Hopkins AR, Zhu L, Yu Q (2019). Efficacy of Proximal Resin Infiltration on Caries Inhibition Results from a 3-Year Randomized Controlled Clinical Trial. J Dent Res.

[9] Carrillo-Díaz M, Migueláñez-Medrán BC, Nieto-Moraleda C, Romero-Maroto M, González-Olmo MJ (2021). How Can We Reduce Dental Fear in Children The Importance of the First Dental Visit. Children (Basel).

[10] Ladewig NM, Tedesco TK, Gimenez T, Braga MM, Raggio DP (2018). Patient-reported outcomes associated with different restorative techniques in pediatric dentistry A systematic review and MTC meta-analysis. PLoS One.

[11] Foster Page LA, Beckett D, Ahmadi R, Schwass DR (2017). Resin Infiltration of Caries in Primary Molars in a Community Setting 24-Month Randomized Controlled Trial Findings. JDR Clin Trans Res.

[12] (2017). Prescribing Dental Radiographs for Infants, Children, Adolescents, and Individuals with Special Health Care Needs. Pediatr Dent.

[13] Mejàre I, Källest IC, Stenlund H (1999). Incidence and progression of approximal caries from 11 to 22 years of age in Sweden A prospective radiographic study. Caries Res.

[14] Howard KE, Freeman R (2007). Reliability and validity of a faces version of the Modified Child Dental Anxiety Scale. Int J Paediatr Dent.

[15] Manoharan V, Arun Kumar S, Arumugam SB, Anand V, Krishnamoorthy S, Methippara JJ (2019). Is Resin Infiltration a Micro invasive Approach to White Lesions of 110 Calcified Tooth Structures : A Systemic Review. Int J Clin Pediatr Dent.

[16] Chen Y, Chen D, Lin H (2021). Infiltration and sealing for managing non-cavitated proximal lesions a systematic review and meta-analysis. BMC Oral Health.

[17] Yang S, Sui B, Cui Y, Liu X, Sun J, Wang J (2022). A novel dental infiltration resin based on isosorbide-derived dimethacrylate with high biocompatibility, hydrolysis resistance, and antibacterial effect. Front Bioeng Biotechnol.

[18] Yu OY, Zhao IS, Mei ML, Lo ECM, Chu CH (2018). Caries-arresting effects of silver diamine fluoride and sodium fluoride on dentine caries lesions. J Dent.

[19] Ayar MK (2016). A review of ethanol wet-bonding Principles and techniques. Eur J Dent.

[20] Li F, Liu XY, Zhang L, Kang JJ, Chen JH (2012). Ethanol-wet bonding technique may enhance the bonding performance of contemporary etch-and-rinse dental adhesives. J Adhes Dent.

[21] Lasfargues JJ, Bonte E, Guerrieri A, Fezzani L (2013). Minimal intervention dentistry part 6. Caries inhibition by resin infiltration. Br Dent J.

[22] Zhou Y, Matin K, Shimada Y, Sumi Y, Tagami J (2017). Evaluation of resin infiltration on demineralized root surface An in vitro study. Dent Mater J.

[23] Shah S, Bhaskar V, Venkatraghavan K, Choudhary P, Ganesh M, Trivedi K (2014). Silver diamine fluoride a review and current applications. J Adv Oral Res.

[24] Hammersmith KJ, DePalo JR, Casamassimo PS, MacLean JK, Peng J (2020). Silver Diamine Fluoride and Fluoride Varnish May Halt Interproximal Caries Progression in the Primary Dentition. J Clin Paediatr Dent.

[25] Paris S, Bitter K, Naumann M, Dörfer CE, Meyer-Lueckel H (2011). Resin infiltration of proximal caries lesions differing in ICDAS codes. Eur J Oral Sci.

[26] Clemens J, Gold J, Chaffin J (2018). Effect and acceptance of silver diamine fluoride treatment on dental caries in primary teeth. J Public Health Dent.

[27] Arthur RA, Zenkner JE, Ornellas Pereira Júnior JC, Correia RT, Alves LS, Maltz M (2018). Proximal carious lesions infiltration-a 3-year follow-up study of a randomized controlled clinical trial. Clin Oral Investig.

[28] Zhi QH, Lo EC, Lin HC (2012). Randomized clinical trial on effectiveness of silver diamine fluoride and glass ionomer in arresting dentine caries in preschool children. J Dent.

[29] Altarabulsi MB, Alkilzy M, Splieth CH (2013). Clinical applicability of resin infiltration for proximal caries. Quintessence Int.

[30] Mattos-Silveira J, Floriano I, Ferreira FR (2014). New proposal of silver diamine fluoride use in arresting approximal caries study protocol for a randomized controlled trial. Trials.

